# Intrasurgical Imaging of Subinternal Limiting Membrane Blood Diffusion in Terson Syndrome

**DOI:** 10.1155/2014/689793

**Published:** 2014-08-14

**Authors:** Lorenzo Iuliano, Giovanni Fogliato, Marco Codenotti

**Affiliations:** Department of Ophthalmology, San Raffaele Scientific Institute, Vita-Salute University, Via Olgettina 60, 20132 Milan, Italy

## Abstract

We report a case of Terson syndrome, providing the first intrasurgical imaging of subinternal limiting membrane blood diffusion in Terson syndrome. We highlight some remarkable in vivo anatomical findings that may give a contribution to the debate about its pathogenesis. Here we hypothesize that the subretinal space might be unlikely to be a primary source of intraocular hemorrhage, and we support the two generally accepted theories about blood diffusion from the retinal vasculature or from the perivascular spaces.

## 1. Introduction

Terson syndrome is a rare condition characterized by subarachnoid hemorrhage in association with intraocular hemorrhage. Terson hemorrhage constitutes a common complication of aneurysmal subarachnoid hemorrhage instances (8–14.5%) and represents 5.5% of all nondiabetic and nontraumatic vitreous hemorrhages [1, 2].

The pathophysiology of Terson syndrome and intraocular blood spreading have been extensively debated in literature [1–3]. Three blood locations in the eye have been described: intravitreal, subinternal limiting membrane (ILM), and subretinal [1].

Here we describe a case report to provide the first intrasurgical imaging of sub-ILM blood diffusion in Terson syndrome and to speculate on its possible pathological mechanisms.

## 2. Case Presentation

A 37-year-old woman was seen in our emergency department because of right subarachnoid and subdural hemispheric hemorrhage. Fundus examination revealed few peripheral little hemorrhages in the right eye and a retrohyaloid hemorrhage in the left eye. Nd:YAG laser membranotomy was performed as first step management, but laser applications failed to perforate the hyaloid, probably due to hardening of the blood clot, and no drainage of blood was achieved. After a one-week observation period, we did not observe any significant change. Hence, a transconjunctival sutureless vitrectomy with posterior hyaloid and ILM peeling was successfully carried out, with full postoperative visual recovery.

**Figure 1:**
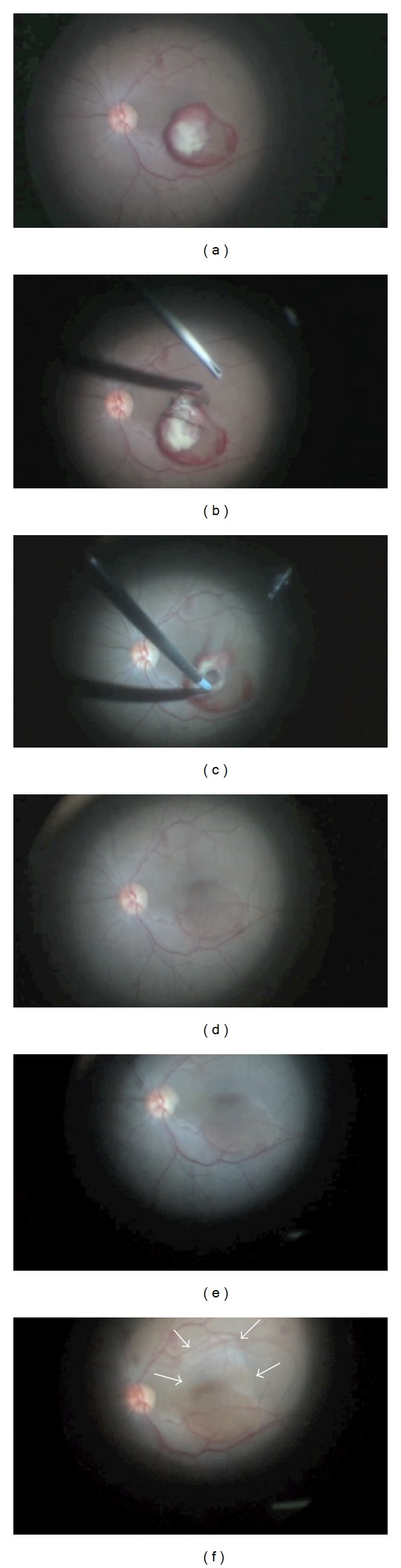
(a) Intact clot after posterior hyaloid peeling; (b) internal limiting membrane (ILM) peeling; (c) gentle suction of sub-ILM hemorrhagic-fibrinoid material; (d) retinal aspect after hemorrhage evacuation; (e) Brilliant Blue staining of the remnant ILM; (f) bleaching of the retinal tissue (white arrows) after enlargement of ILM peeling out of hemorrhage region edges.

The digital video of the whole surgery procedure was carefully reviewed. Posterior hyaloid peeling left the clot intact (Figure 1(a)). Partial removal of the clot was obtained with ILM peeling, which allowed the blood to flow out into the vitreous cavity (Figure 1(b)). Gentle suction provided total evacuation of sub-ILM hemorrhagic-fibrinoid material (Figure 1(c)). Brilliant Blue (Geuder AG, Heidelberg, Germany) assisted ILM peeling enlargement out of the edges of the ILM-peeled hemorrhage area showed marked bleaching of the surrounding retinal tissue (Figure 1(f), white arrows), indicating absence of blood beneath the retinal tissue uninvolved by hemorrhage.

## 3. Discussion

This case unexpectedly demonstrates the presence of sub-ILM and also signs of intraretinal blood. This finding is in agreement with Morris and colleagues' classification, which histologically describes the hemorrhage potential locations. The authors distinguish indeed between “submembranous hemorrhagic macular cyst” (sub-ILM) and “preretinal hemorrhagic macular cyst” (between ILM and posterior hyaloid) [2].

Unfortunately, blood location does not unquestionably suggest the blood entrance mechanisms. Two theories are generally accepted to describe the pathogenetic process of Terson syndrome: blood diffusing directly into the vitreous cavity from the optic disc area and blood spreading under the ILM or subhyaloid from retinal venous arcades caused by elevated intracranial pressure (outflow blockade) [3, 4]. As suggested in literature [1], both theories may be correct and complement one another. Indeed, blood could initially enter one compartment and diffuse to another or enter directly two or more compartments (sub-ILM, subhyaloid, or intravitreal). Furthermore, a recent research by Sakamoto and colleagues reported that the subarachnoid hemorrhage within the optic nerve sheath may enter beneath the ILM through the perivascular space surrounding retinal vessels (Virchow-Robin spaces) [5].

Our case report highlights that the transretinal or the intraretinal blood spreading is minimal and confined in Terson syndrome, as documented by our figures. Hence, we hypothesize that the subretinal space might be unlikely to be a primary source of intraocular hemorrhage or a blood-spreading site in Terson syndrome [1]. We emphasize the importance of the two theories about blood diffusion sub-ILM or subhyaloid from the retinal vasculature or from the perivascular spaces.
